# The Role of Non-Peripancreatic Lymph Nodes in the Survival of Patients Suffering from Pancreatic Cancer of the Body and Tail: A Systematic Review and Meta-Analysis of High-Quality Studies

**DOI:** 10.3390/cancers15082322

**Published:** 2023-04-16

**Authors:** Stefano Granieri, Alessia Kersik, Alessandro Bonomi, Simone Frassini, Davide Bernasconi, Sissi Paleino, Alessandro Germini, Elson Gjoni, Christian Cotsoglou

**Affiliations:** 1 General Surgery Unit, ASST-Brianza, Vimercate Hospital, Via Santi Cosma e Damiano, 10, 20871 Vimercate, Italy; 2 General Surgery Residency Program, University of Milan, Via Festa del Perdono, 7, 20122 Milan, Italy; 3 General Surgery Residency Program, University of Pavia, Corso Str. Nuova, 65, 27100 Pavia, Italy; 4 General Surgery Unit, Department of Surgery, Fondazione I.R.C.C.S. Policlinico San Matteo, Viale Camillo Golgi, 19, 27100 Pavia, Italy; 5 Center of Biostatistics for Clinical Epidemiology, University of Milan—Bicocca, Via Cadore, 48, 20854 Monza, Italy

**Keywords:** pancreatic cancer of the body and tail, pancreatic neoplasm, lymphadenectomy, lymph node excision, systematic review, meta-analysis

## Abstract

**Simple Summary:**

Lymph nodes metastases represent a well-known negative prognostic factor for patients suffering from pancreatic cancer of the body and tail. To date, international guidelines do not recommend an extended lymphadenectomy in left-sided pancreatic resections. However, the extent of the lymphadenectomy in these cases is still debated. The present study aimed to systematically review the current literature to explore the incidence and the prognostic impact of non-peripancreatic lymph nodes in patients suffering from pancreatic cancer of the body and tail. After the literature review, eight studies were included in the meta-analysis. An increased risk of death for patients with positive non-PLNs was detected (HR: 2.97; 95% CI: 1.81–4.91; *p* < 0.0001). Meta-analysis of the proportions highlighted different proportions of nodal infiltration according to nodal stations. A systematic extended lymphadenectomy, despite its potential beneficial prognostic effect, could not be recommended yet based on the results of the present study.

**Abstract:**

Lymph nodes (LNs)’ metastases have a well-known detrimental impact on the survival outcomes of patients suffering from pancreatic cancer of the body and tail. However, the extent of the lymphadenectomy for this tumor location is still debated. The aim of this study was to systematically review the current literature to explore the incidence and the prognostic impact of non-peripancreatic lymph nodes (PLNs) in patients suffering from pancreatic cancer of the body and tail. A systematic review was conducted according to PRISMA and MOOSE guidelines. The primary endpoint was to assess the impact of non-PLNs on overall survival (OS). As a secondary endpoint, the pooled frequencies of different non-PLN stations’ metastatic patterns according to tumor location were explored. Eight studies were included in data synthesis. An increased risk of death for patients with positive non-PLNs was detected (HR: 2.97; 95% CI: 1.81–4.91; *p* < 0.0001). Meta-analysis of proportions pointed out a 7.1% pooled proportion of nodal infiltration in stations 8–9. The pooled frequency for station 12 metastasis was 4.8%. LN stations 14–15 were involved in 11.4% of cases, whereas station 16 represented a site of metastasis in 11.5% of cases. Despite its potential beneficial effect on survival outcome, a systematic extended lymphadenectomy could not be recommended yet for patients suffering from PDAC of the body/tail.

## 1. Introduction

Pancreatic ductal adenocarcinoma (PDAC) is a relatively uncommon tumor in the general population, but it represents the 7th leading cause of cancer death, accounting for approximately 49,000 estimated deaths worldwide in 2020 [1,2]. Although less frequent compared to cancers of the head or of the pancreas, PDAC of the body/tail has shown an increasing incidence rate over recent decades [3].

The presence of lymph node (LN) metastases represents a well-known detrimental prognostic factor for both overall and disease-free survival [4,5]. Nevertheless, an extended lymphadenectomy for PDAC arising in the pancreatic head has not been demonstrated to be effective in improving survival outcomes [6,7,8,9,10]. Indeed, the 2014 International Study Group of Pancreatic Surgery (ISGPS) consensus statement, as well as other international guidelines, does not recommend an extended lymphadenectomy while performing pancreatoduodenectomy [11,12,13,14]. On the other hand, a standard lymphadenectomy encompassing only stations 10, 11, and 18 is recommended for PDACs of the body/tail, with station 9 only suggested to be harvested in the presence of tumors confined to the area of the body of the pancreas. However, a substantial lack of reliable evidence burdens the strength of this recommendation. As a matter of fact, the extent of the lymphadenectomy in the tumors arising in the pancreatic body and tail is still debated. The scope of the present study is to systematically review the current literature to explore the incidence and the prognostic impact of non-peripancreatic lymph nodes (PLNs) on patients suffering from pancreatic cancer of the body and tail.

## 2. Materials and Methods

### 2.1. Search Strategy

The protocol for the present systematic review was registered on PROSPERO and can be accessed using the following ID: CRD42022341062. The Preferred Reporting Items for Systematic Reviews and Meta-Analyses (PRISMA) and the Assessing the Methodological Quality of Systematic Reviews (AMSTAR II) guidelines [15,16] were followed.

The PubMed, Scopus, and Cochrane Library databases were screened without time restrictions up to 30 May 2022. The research also included all of the MeSH terms. The full search queries are available in Supplementary Materials.

Articles without free full text available were searched through the University of Milan digital library, the “Alberto Malliani” library, or via direct contact with the authors. A search of references of included studies and previous reviews on the topic was also performed by hand to include additional relevant studies according to our selection criteria. Two investigators (A.K. and A.B.) carried out the literature search independently.

### 2.2. Inclusion Criteria

All study designs were considered. A specific population (P), intervention (I), comparator (C), outcome (O), and study design (S) (PICOS) framework was specified to define study eligibility, as recommended. In detail:

- Population (P): patients suffering from pancreatic ductal adenocarcinoma undergoing distal pancreatosplenectomy with lymphadenectomy;
- Intervention (I): lymphadenectomy including non-PLN stations according to the ISGPS (stations 10–11–18);
- Comparison I: lymphadenectomy involving only PLN stations according to the ISGPS (stations 10–11–18); this criterion was not mandatory;
- Outcomes (O): overall mortality;
- Study design (S): all study designs.
- Studies with insufficient reporting of the PICOS criteria were excluded.

### 2.3. Exclusion Criteria and Quality Assessment of Included Studies

The studies with the following criteria were deemed non-eligible:

(1) Studies also including patients suffering from PDAC of the head without reporting separate data;
(2) Studies reporting overlapping series;
(3) Studies with a less than 3-year follow-up period;
(4) Non-English language papers;
(5) Case reports, editorials, abstracts, unpublished studies, book chapters, and commentaries;
(6) Previously published reviews.

One author (SF) assessed the methodological quality of each retrospective comparative study using the validated Newcastle–Ottawa Scale (NOS) [17].

### 2.4. Systematic Review Process and Data Extraction

Overall, 3013 articles were preliminarily identified in the literature search. After excluding duplicates, two independent reviewers (A.K. and A.B.) screened titles and abstracts of 2242 records using a screening form developed a priori. Investigators were blinded to each other’s decisions. Eventual disagreement was solved through discussion.

**Figure 1:**
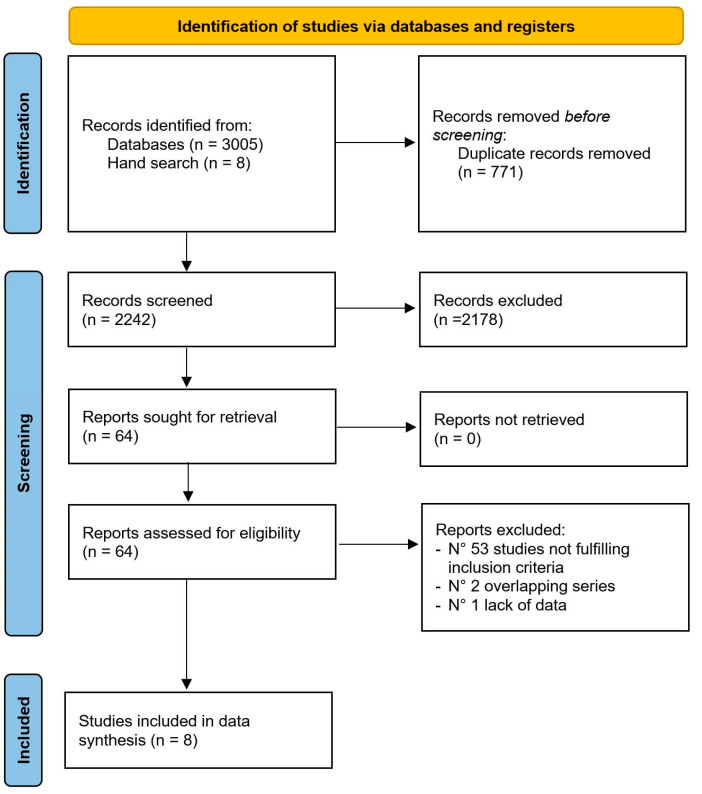
PRISMA flow diagram.

Sixty-four articles were assessed for eligibility, and eight were included in data synthesis. The flowchart depicting the overall review process according to PRISMA is shown in Figure 1.

Data were extracted independently by three authors (S.G., A.K., and A.B.). Information about study design and methodology, participant demographics and baseline characteristics, and survival outcomes were gathered in a computerized spreadsheet (Microsoft Excel 2016; Microsoft Corporation, Redmond, WA, USA). When disagreement arose, two further investigators (A.G. and E.G.) helped resolve it through discussion.

### 2.5. Assessment of Risk of Bias

The risk of bias was assessed for individual studies according to the ROBINS-I tool provided by the Cochrane Collaboration [18] (SF). The following domains were explored: (1) bias arising from the randomization process; (2) bias due to deviations from intended interventions; (3) bias due to missing outcome data; (4) bias in the measurement of the outcome; (5) bias in the selection of the reported results.

Data were collected according to the methodology proposed by Higgins [19] in a computerized spreadsheet. Bar and traffic light plots were created to display the results of the risk of bias assessment graphically.

### 2.6. Endpoints

The primary endpoint was to assess the impact of non-PLN involvement on overall survival (OS). As a secondary endpoint, we explored the pooled frequencies of different non-PLN stations’ metastatic patterns according to tumor location (body, body and tail, and tail of the pancreas).

### 2.7. Statistical Analysis

Hazard Ratio (HR) and 95% Confidence Intervals (CI) represented the primary outcome measures. The secondary outcome measure was represented by the pooled proportion of metastatic LNs for each non-peripancreatic station according to tumor location.

A random effects model based on the generic inverse variance method was built to assess the impact of heterogeneity on the results. Between-studies heterogeneity was quantified using the I^2^ statistic and Cochran’s Q test; cut-off values of 25%, 50%, and 75% were considered low, moderate, and high, respectively [20]. The presence of outliers and/or studies overly contributing to heterogeneity was investigated through the leave-one-out method. Forest plots were developed to display the results graphically.

HRs were retrieved from each manuscript, and their standard errors (S.E.) were computed from the reported 95% confidence intervals (95% CI). If not overtly reported, they were estimated according to the method proposed by Tierney [21] whenever possible. HR and S.E. logarithmic transformation were derived to estimate treatment effects.

Funnel plots were developed to explore publication bias, and Egger’s test of the intercept was used to quantify the funnel plots’ asymmetry.

Statistical analysis was conducted with R statistical software (The Comprehensive R Archive Network—CRAN, ver. 4.0.0 x64) [22] using the “meta”, “metafor”, “robvis”, and “dmetar” packages [23,24,25].

## 3. Results

### 3.1. Descriptive Noncomparative Analysis of Included Studies and Primary Endpoint

After the literature search, eight studies were included in the quantitative analysis [26,27,28,29,30,31,32]. Only five of these reported information regarding the primary endpoint [27,28,31,32,33], whereas three reported data only about the secondary endpoint [26,30].

In total, 869 patients were included in the meta-analysis, comprising 584 for the primary and 285 for the secondary endpoint. All studies scored a NOS ≥ 7. Eight studies were conducted in Eastern countries (Japan, China), and only one was conducted in Italy. All included studies were retrospective.

About the study by Imamura et al. [30], only data regarding tumors located in the body or tail of the pancreas were considered. Despite the study by Hirashita et al. [34] that described the frequency of positive LNs, it was excluded from the secondary endpoint analysis due to the lack of a clear proportion of positive LNs over harvested LNs for each nodal station. This data could not be retrieved through direct contact with the author. The studies by Nakao et al. and Kanda et al. [35,36] were originally considered for qualitative synthesis, but they were excluded because they overlapped with the series reported in the study by Sahin et al. [27]

Further details of the included studies are reported in Table 1.

### 3.2. Primary Endpoint

Generic inverse variance meta-analysis detected a more than twofold increase in the risk of death for patients with positive non-PLNs (HR: 2.35; 95% CI: 1.22–4.50; *p* = 0.01; I^2^: 54.9%).

**Figure 2:**
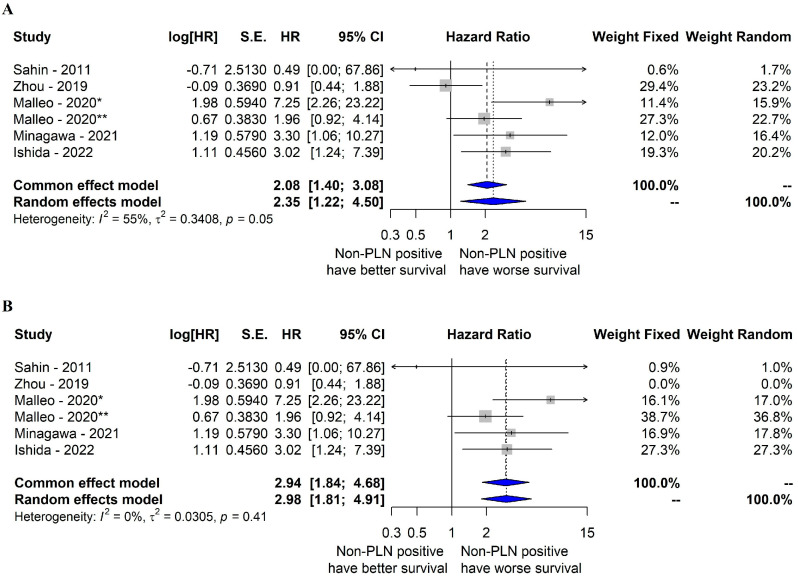
Meta-analysis of generic inverse variance: mortality. Forest plots (**A**) before and (**B**) after sensitivity analysis. * LN station 9; ** LN station 8.

Sensitivity analysis identified the study by Zhou et al. as the only one overtly contributing to heterogeneity (Supplementary Materials Figure S1). After excluding it, a significantly augmented risk of mortality was confirmed for patients with non-PLN involvement (HR: 2.97; 95% CI: 1.81–4.91; *p* < 0.0001; I^2^: 0%). Forest plots before and after sensitivity analysis are displayed in Figure 2.

### 3.3. Secondary Endpoint

**Figure 3:**
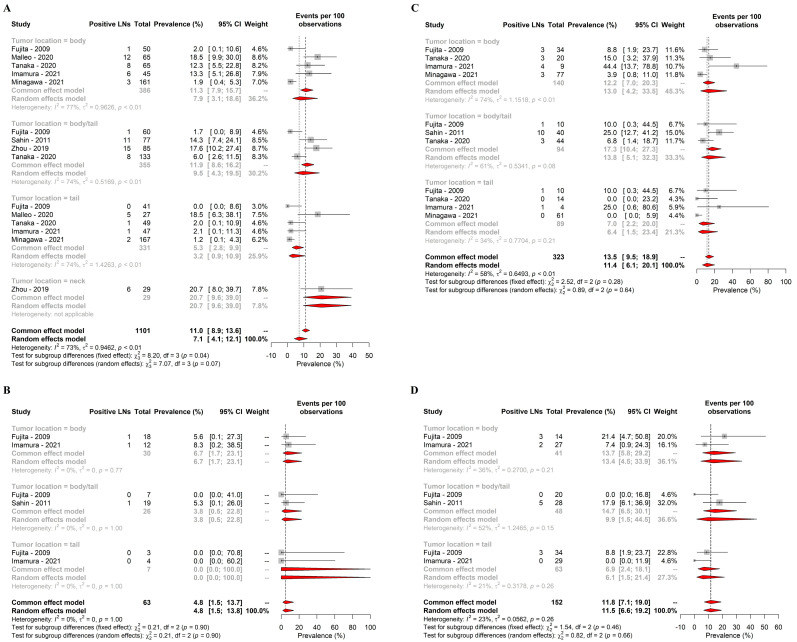
Meta-analysis of proportions: prevalence of nodal metastases to (**A**) stations 8–9; (**B**) station 12; (**C**) stations 14–15; (**D**) station 16.

The meta-analysis of proportions highlighted a 7.1% (six studies [26,27,28,29,30,31]; 95% CI: 4.1%–12.1%; I^2^ = 73.5%) pooled frequency of positive nodal stations 8–9. The greatest proportion was noted for the subgroup of patients with tumors located in the neck of the pancreas (20.7%) (Figure 3A).

The pooled proportion of metastatic LNs in station 12 was 4.8% (three studies [26,27,30]; 95% CI: 1.5%–13.8%; I^2^ = 0%). The greatest proportion was noted for the subgroup of patients with tumors located in the body of the pancreas (6.7%) (Figure 3B).

LN stations 14–15 were involved in 11.4% of cases (five studies [26,27,29,30,31]; 95% CI: 46.1–20.1%; I^2^ = 58%). The highest prevalence of metastasis to these nodal stations was observed for tumors located in the pancreatic body/tail (11.3%) (Figure 3C).

Station 16 represented a site of metastasis in 11.5% of cases (three studies [26,27,30]; 95% CI: 6.6%–19.2%; I^2^ = 22.5%). The highest prevalence of metastasis to these nodal stations was observed for tumors located in the body (13.4%) (Figure 3D).

### 3.4. Quality of the Studies and Risk of Bias Assessment

**Figure 4:**
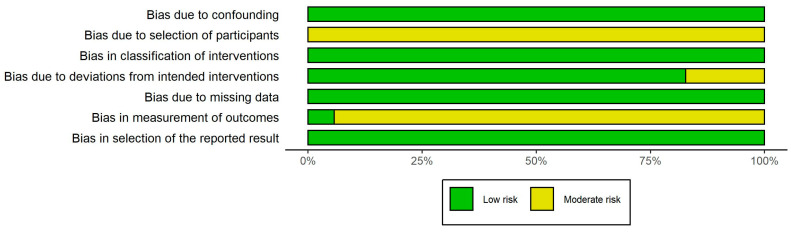
Risk of bias assessment summarized in a bar plot.

Five studies scored a NOS of seven points [26,27,30,31,32], whereas for the remaining three, it was eight [28,29,33]. Figure 4 summarizes the risk of bias evaluation according to the latest version of the Cochrane Collaboration handbook [19]. None of the studies were burdened by a severe risk of bias. Bias due to the selection of participants and in measurement of outcomes were the two domains accounting for most of the risk of bias observed. Figure 4 summarizes RoB assessment with a bar plot. More detailed information is displayed in the traffic light plot reported in Supplementary Materials Figure S2.

### 3.5. Assessment of Publication Bias

**Figure 5:**
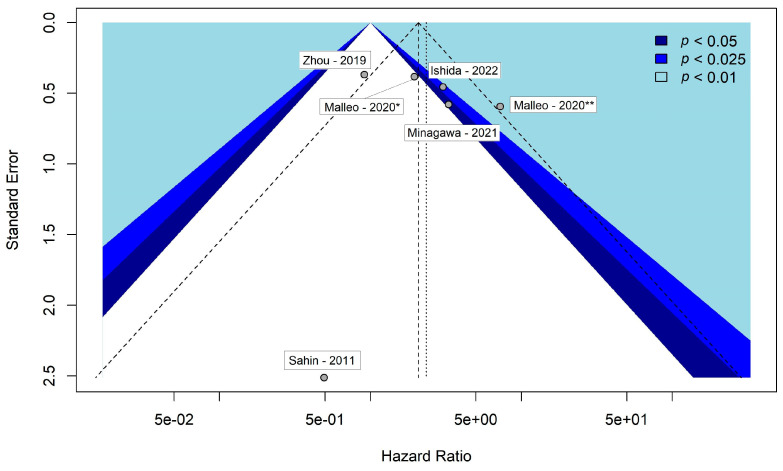
Contour-enhanced funnel plot of publication bias. * LN station 9; ** LN station 8.

According to the funnel plot (Figure 5) and Egger’s test (*p* = 0.64), the presence of publication bias for the primary endpoint was ruled out. However, this evaluation is doubtful due to the small number of studies included in the analysis.

## 4. Discussion

The extent of lymph node dissection in pancreatic cancer of the body/tail still represents a matter of debate. The 2014 ISGPS consensus conference recommendation for patients with left-sided pancreatic malignancies encompasses a standard station 10, 11, and 18 lymphadenectomy. The harvesting of station 9 is suggested only in the presence of tumors arising from the pancreatic body [11]. In this regard, the study by Zhou et al. highlighted that station 9 was involved in 20.7% of PDACs of the neck and in 17.6% of the tumors located in the body/tail of the gland [33]. However, survival analysis failed to demonstrate a benefit for patients with station 9 free from metastasis, but the authors concluded that station 9′s involvement not only should not be considered a contraindication to curative surgery but also should be routinely included in lymphadenectomy.

On the other hand, the Japanese Pancreatic Society (JPS) currently recommends against prophylactic extended lymph node and nerve plexus dissection due to the lack of improvement in survival outcomes [38]. Nevertheless, the literature regarding this topic is made exclusively of retrospective evidence, and to date, there are no ongoing RCTs exploring the efficacy on survival of standard vs. extended lymphadenectomy.

Currently, only the study published by Lee et al. in 2017 claimed a survival advantage for patients suffering from PDAC of the body/tail undergoing extended LN dissection [39]. In this study, 59 patients with pancreatic body cancer and 48 with pancreatic tail cancer were included. Eighty-five percent of patients with pancreatic body cancer and sixty-three percent with pancreatic tail cancer received extended lymphadenectomy. The authors concluded that patients with pancreatic body cancer (but not with pancreatic tail cancer) who underwent extended lymphadenectomy had significantly greater disease-free incidence and overall survival compared to those receiving only peripancreatic LN dissection. Nevertheless, the results of this study should be interpreted with caution due to its retrospective nature, the sample size imbalance between the two groups of patients (with only nine patients receiving standard lymphadenectomy), and the limited median follow-up (17 months).

The absolute number of LNs harvested during both proximal and distal pancreatic resection has historically represented a well-known prognostic factor for pancreatic cancer patients. However, because nodal disease per se has not always been demonstrated as a significant predictor, its clinical impact on survival outcomes has progressively been challenged in favor of the more comprehensive concept of lymph node ratio (LNR) [40,41].

A multitude of cornerstone studies demonstrate how LNR performed way better compared to nodal disease alone (N0/N+) and the absolute number of LNs retrieved in the stratification of prognosis, also in the subgroup of node-positive patients [41,42,43,44]. The results of the aforementioned trials helped to establish a LNR of 0.2 as the optimal cutoff value for this purpose.

However, because LNR represents the ratio between the number of LN involved and examined lymph nodes (ELN), it should be kept in mind that this latter parameter depends on multiple variables including the anatomic constitution of every patient, the extent of surgery, and the technique of pathological examination. In this regard, a recent study by Malleo et al. pointed out that the minimum number of ELN to ensure an appropriate nodal staging during resection of left-sided PDACs should be 20. Such a threshold was demonstrated to have a strong prognostic impact, mainly in node-negative patients, with a threefold increase in disease-related mortality for those with <20 ELN [45].

In this scenario, one could argue that the more LNs retrieved, the better the nodal staging and survival outcomes that can be reached. The antegrade modular approach to left-sided pancreatosplenectomy (RAMPS), first introduced almost 20 years ago, has gained success over recent years, and a solid body of evidence advocates a survival advantage mainly related to its ability to excise more LNs. Conversely, the most recent meta-analysis by Watanabe et al. suggests that, despite the fact that this technique allows the harvesting of a significantly greater number of LNs compared to standard distal pancreatectomy, there is no clear benefit in terms of overall and recurrence-free survival [46]. Nevertheless, it must be noted that the aforementioned meta-analysis, even though more correct from a methodological point of view compared to previous ones, was based almost exclusively on retrospective, non-matched studies, of which only four out of thirteen scored ≥ 7 on the Newcastle–Ottawa scale. The authors themselves recognized these limitations underlined the low-grade certainty of the evidence, and they concluded that RAMPS has little effect on the prognoses of patients with left-sided pancreatic cancer. This reinforces the need for further well-designed and randomized studies.

It has been suggested that when a higher number of LNs is examined, the number of PLNs outperforms the LNR in predicting survival outcomes [5,47]. However, if this assumption had been widely proven right for proximal pancreatic resections, not as many scientific reports are available in the current literature exploring the prognostic impact of different LN parameters in left-sided pancreatic resections.

In recent years, many studies, especially the ones included in our meta-analysis, brought to attention the concept that left-sided PDACs do not have all of the same lymphatic drainage. Body tumors likely have different drainage to the celiac axis and SMA nodes compared to tumors located in the tail. That said, thinking about the most likely drainage and performing the most appropriate lymphadenectomy based on the primary tumor location should be kept in mind during distal pancreatectomy. In this scenario, the present study emphasizes the detrimental impact on the survival of positive non-peripancreatic LN stations. Our results highlight a threefold increase in the risk of death for patients experiencing metastases in non-PLNs, with different pooled frequencies of nodal involvement according to tumor location. Nevertheless, it is worth noting that, regarding the primary endpoint, only two studies [28,33] explored the role of station 9, one [31] explored the mixed effect of stations 8 and 14, and two [27,32] explored the mixed effect of all non-PLNs. As such, this limit contributes to increasing the heterogeneity that burdens our results. Among the studies included, only three conducted multivariate analysis to explore the independent effects of specific LN stations’ infiltration on survival [28,31,32]. Of these, the study by Malleo et al. was the one reporting the highest HR for non-PLN infiltration, specifically for station 9 (HR: 7.25), whereas Minagawa et al. detected a more than threefold increase in the risk of death for patients with stations 8 or 14 involved (HR: 3.3).

Some further careful considerations should be done. LN stations 14–15 and 16 were the most frequently involved; for stations 14–15, the highest prevalence of metastasis was noted for tumors arising from the pancreatic body/tail (13.8%). On the opposite side, stations 8–9 and 12 were infiltrated only in 7.1% and 4.8% of cases, respectively, but most of the nodal metastases were observed for PDACs of the neck (20.7%) and body (6.7%). It is noteworthy that meta-analyses of proportions for stations 8–9 and 14–15 were burdened by high between-study heterogeneity. On the other hand, the analysis of stations 12 and 16 was based on the pooled estimates of only three studies.

Several reports recently brought to attention the relationship between a tumor’s location and its nodal involvement, mainly focusing on the anatomic position of the neoplasm (pancreatic neck, body, or tail) [29,30,34]. In 2019, Zhou et al. explored how the distance of the pancreatic tumor from the porto-mesenteric confluence (margin-to-bifurcation distance, or MTBD) impacted station 9 involvement. They found that tumors arising in the pancreatic neck with a MTBD ≤ 2.5 cm had station 9 LN metastases in 17.6% of cases; conversely, when MTBD was > 2.5 cm, station 9 invasion was observed only in 1.8% of patients. Similarly, in 2022, Ishida et al. underlined the importance of this concept by evaluating the distance of the tumor from the left side of the portal vein (DPT) to predict the probability of non-PLN involvement. The authors found that patients with non-PLN positive LNs had the tumor significantly closer to the left margin of the portal vein compared with those with only-PLN positive LNs (5 vs. 28 mm, *p* = 0.006), and non-PLN metastases were observed exclusively in patients with DPT < 20 mm. Indeed, the authors identified through ROC curve analysis the optimal DPT cutoff value (19 mm) to predict the risk of non-PLN involvement. This data not only suggests a linear relationship between the tumor location and the risk of nodal involvement, but also that a quantitative measure of the tumor distance from the portal vein/porto-mesenteric confluence may be helpful in tailoring the extent of nodal dissection.

To the best of our knowledge, the present study represents the first systematic review of the literature with meta-analysis about the prognostic implications of nodal metastases in left-sided pancreatic cancer patients. It specifically focuses on the impact of non-PLN on prognosis and explores in-depth the frequency of nodal metastases to specific stations as well. All studies included in the quantitative analysis scored ≥ 7 on the Newcastle–Ottawa scale, and none of them were burdened by severe risk of bias.

Nevertheless, some non-negligible limitations burden the results of our study, the first being the retrospective nature of the studies included. Furthermore, the number of studies eligible for data synthesis was quite low. Under a more “statistical” point of view, besides between-studies heterogeneity, it should also be underlined that some effect sizes (HRs) were derived from multivariate analysis [28,31,32], whereas some others came from univariate analysis [28] or were extracted from Kaplan–Meier curves [27,33]. Finally, it should be noted that in the present meta-analysis, the effects of confounding variables (e.g., resection margins, adjuvant treatments, biological and molecular profiling of the tumor, etc.) on survival outcomes were not explored.

## 5. Conclusions

The results of the present study underline the significatively detrimental effect of metastatic non-PLN on the prognosis of patients suffering from PDAC of the body and tail. In light of the characteristics of the studies included and the quality of the results obtained, a systematic extended lymphadenectomy could not be recommended yet for these patients, despite its potentially beneficial effect on survival outcomes. Our study emphasizes the urgent need for reliable and quality evidence from randomized controlled trials to better define the optimal extent of LN dissection in left-sided pancreatic cancer.

## Figures and Tables

**Table 1:** Characteristics of included studies. NS: not specified; * computed using the quantile estimation (QE) method [37].

Author	Year of Publication	Years of Enrollment	Country	NOS	Age	N° of Pts	LN Stations Involved in Lymphadenectomy		LN Stations Definition	Tumor Location
Fujita [26]	2009	1993–2008	Japan	7	64.2 (40–81) (Mean—range)	50	8-9-10-11-12-13-14-16-17-18		JPS	50% body; 34% body/tail; 16% tail
Sahin [27]	2011	1991–2010	Japan	7	63 (38–79) (Median—range)	85	8-10-11-18 (adjacent LNs)	9-12-13-14-15-16-17 (distant LNs)	JPS	82.3% body; 17.7% tail
Zhou [33]	2019	2013–2016	China	8	NS	169	9-10-11-18 (standard LND)	8-14-16 (extended LND)	ISGPS	17.2% neck; 82.8% body/tail
Malleo [28]	2020	2001–2017	Italy	8	68 (59–74) (Median—IQR)	100	8-10-11-18 (+9 in selected cases)		ISGPS	69% body; 31% tail
Tanaka [29]	2020	2002–2019	Japan	8	69 (44–85) (Median—range)	100	8-9-10-11-14-16-18		JPS	23% body; 52% body/tail; 25% tail
Imamura [30]	2021	2002–2015	Japan	7	68 (67–70) (Median—IQR) *	135	8-9-10-11-12-13-14-16-18		AJCC	44% body; 66% tail
Minagawa [31]	2021	2007–2018	Japan	7	71 (68–73) (Median—IQR) *	120	8-9-10-11-18 (+14a for PDAC of the body)		ISGPS	48.3% body; 51.7% tail
Ishida [32]	2022	2007–2020	Japan	7	73 (47–89) (Median—range)	110	7-8-9-10-11-14-18		JPS	NR

## Data Availability

Data are available from the corresponding author under reasonable request.

## Supplementary Materials

The following supporting information can be downloaded at: https://www.mdpi.com/article/10.3390/cancers15082322/s1; Search queries; Figure S1: Sensitivity analysis primary endpoint; Figure S2: Risk of bias assessment: traffic light plot, PRISMA checklist; AMSTAR II checklist.
