# Supplementary material for: The Role of Non-Peripancreatic Lymph Nodes in the Survival of Patients Suffering from Pancreatic Cancer of the Body and Tail: A Systematic Review and Meta-Analysis of High-Quality Studies
Source: Cancers (Basel). 2023 Apr 16;15(8):2322. doi: 10.3390/cancers15082322 (PMC10136481; doi:10.3390/cancers15082322)
Supplement: Supplementary file 1 [file cancers-15-02322-s001.zip › cancers-2265056-SI.pdf]

The role of non-peripancreatic lymph nodes involvement on survival in patients suffering from pancreatic cancer of the body and tail. A systematic review and meta-analysis

SUPPLEMENTARY MATERIALS

Index

1) Search queries	2
2) Sensitivity analysis primary endpoint	3
3) Risk of bias assessment: traffic light plot	4

1) **Search queries**

Pubmed and Cochrane Library

((distal pancreatectomy) OR (left pancreatectomy) OR (pancreaticosplenectomy) OR (pancreatosplenectomy) OR (RAMPS) OR (radical antegrade modular pancreatosplenectomy)) AND ((lymphadenectomy) OR (extended lymphadenectomy) OR (lymph nodes) OR (lymph node removal) OR (lymph node harvesting) OR (nodes dissection)) AND ((survival) OR (overall survival) OR (disease free survival) OR (progression free survival) OR (hazard ratio))

Scopus

("distal pancreatectomy" OR "left pancreatectomy" OR "pancreaticosplenectomy" OR "pancreatosplenectomy" OR "RAMPS" OR "radical antegrade modular pancreatosplenectomy") AND ("lymphadenectomy" OR "extended lymphadenectomy" OR "lymph nodes" OR "lymph node removal" OR "lymph node harvesting" OR "nodes dissection") AND ("survival" OR "overall survival" OR "disease free survival" OR "progression free survival" OR "hazard ratio")

2) **Sensitivity analysis primary endpoint**

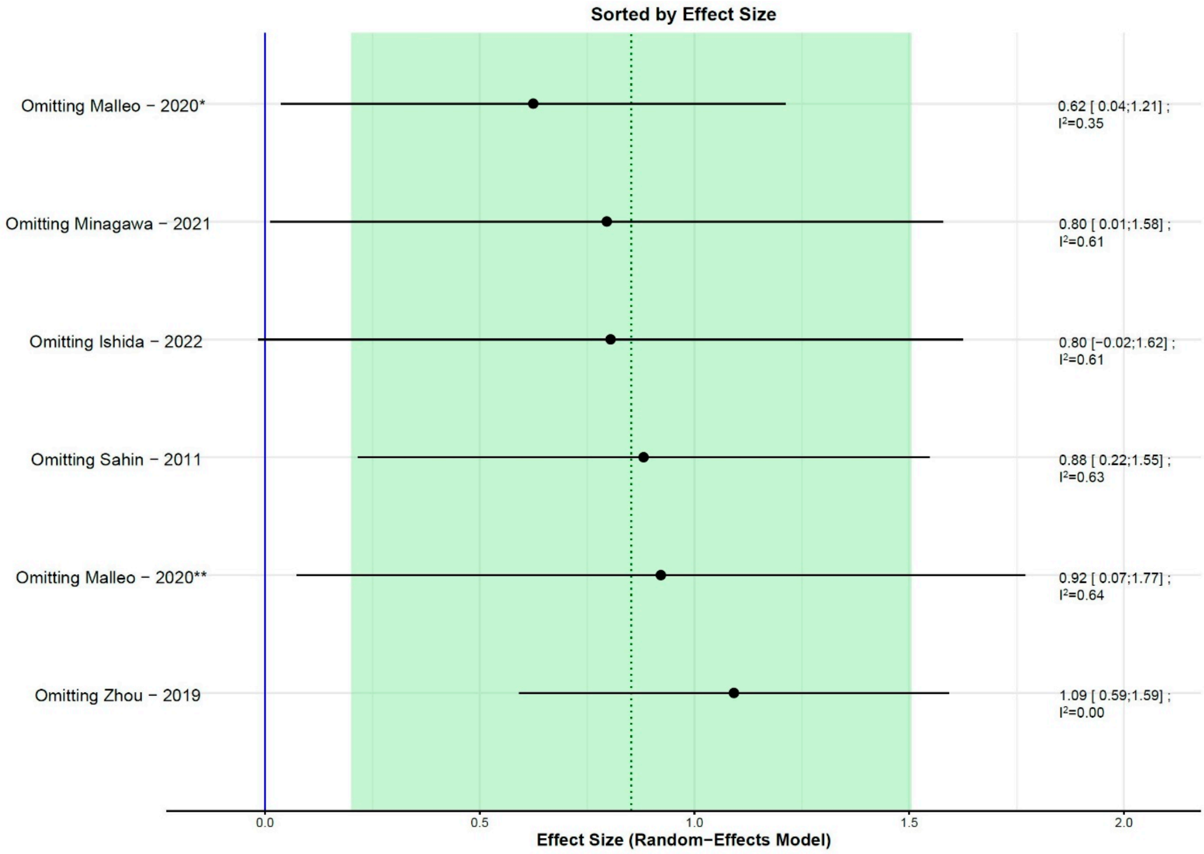

Figure S1: sensitivity analysis through leave-one-out method

3) Risk of bias assessment: traffic light plot

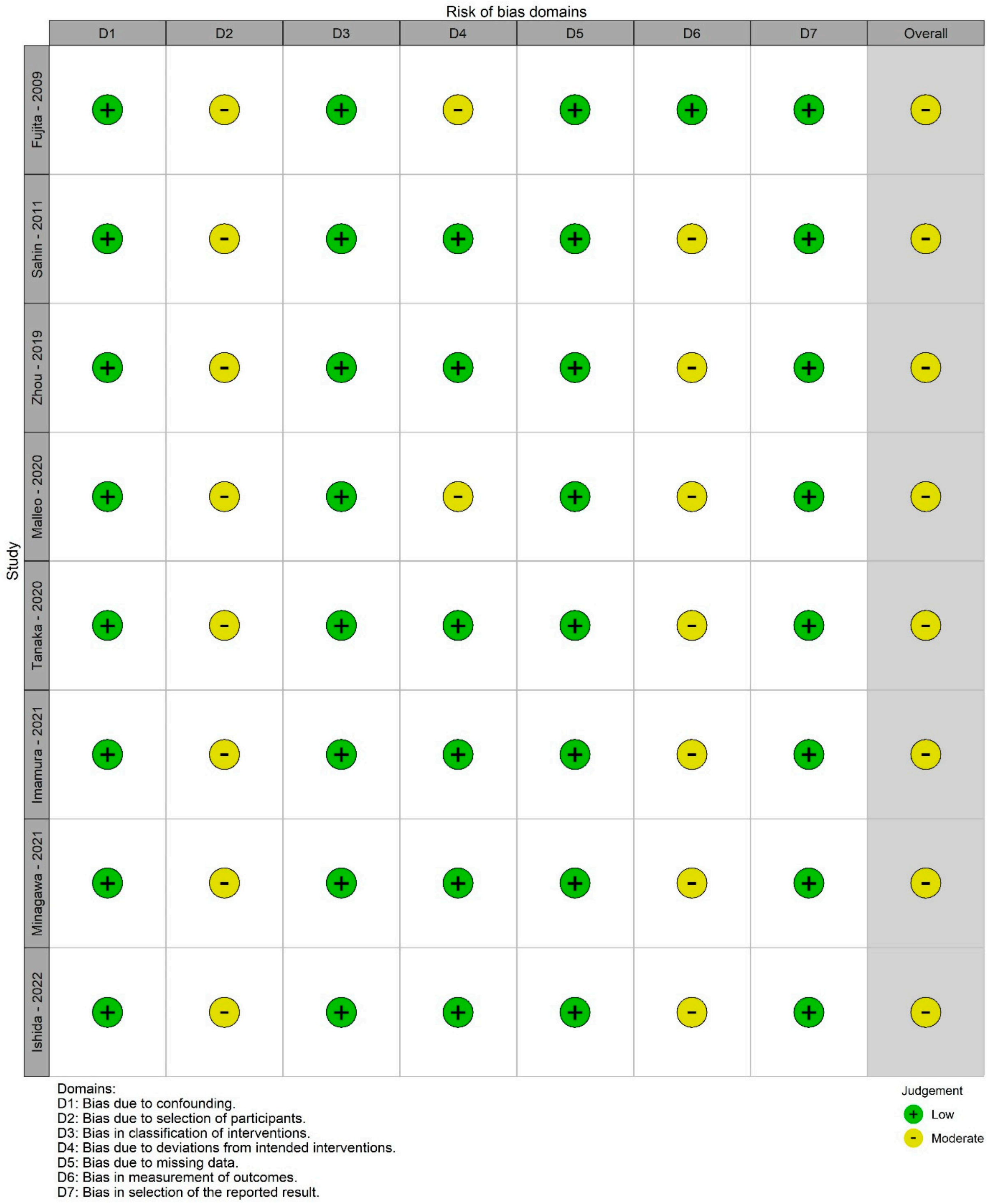

Figure S2: Risk of bias assessment - traffic light plot
